# Validation of national hospital antimicrobial consumption data in England

**DOI:** 10.1186/2047-2994-4-S1-P172

**Published:** 2015-06-16

**Authors:** B Muller-Pebody, D Ladenheim, C Fuller, D Ashiru-Oredope, S Hopkins

**Affiliations:** 1 HCAI & AMR, CIDSC Public Health England, UK; 2 Health Expert Advisory Committee on AMR & HCAI (ARHAI), Public Health England , London, UK

## Introduction

In 2014, the first national report providing surveillance data for antibiotic consumption in England was published by the English Surveillance Programme for Antimicrobial Utilisation and Resistance (ESPAUR) [1].

Rx-info and IMS Health are commercial organisations specialising in the provision of prescribing data for healthcare. Data from IMS Health was used to collate information on antibiotic use in secondary care for the ESPAUR report. The report showed considerable variability in the extent of antibiotic prescribing across different areas within England. While IMS Health and Rx-info each have formal internal quality assurance processes, there has been no external validation of the data when used for the purpose of comparison or benchmarking. The National Health Service (NHS) England will include validation of hospital prescribing data as part of the antimicrobial quality premium (financial reward for improvements in quality of care) in 2015/16.

## Objectives

To develop a national protocol for validating antimicrobial consumption data in acute NHS hospitals in England.

## Methods

A validation protocol was designed and piloted in acute NHS hospitals. Each was sent the protocol along with a workbook to enable each hospital to submit antimicrobial consumption data from their own pharmacy system. These data were analysed alongside that from IMS Health and Rx-info. Participating hospitals were also sent a questionnaire to gather feedback on the pilot validation protocol to help inform the final national protocol.

## Results

Forty-five (out of acute 156) NHS Trusts participated in the pilot. The pilot protocol and questionnaire feedback enabled the development of a national validation protocol which was published in March 2015.

## Conclusion

All acute NHS hospitals in England will be encouraged to use the national protocol to validate antimicrobial prescribing data to ensure correct and meaningful data for the purpose of benchmarking and antibiotic stewardship.

## Disclosure of interest

None declared.

## References

[B1] Public Health England English surveillance programme antimicrobial utilisation and resistance (ESPAUR) report - Publications - GOV.UK [Internet] 2014 [cited 2014 Dec 30]. Available from: https://www.gov.uk/government/publications/english-surveillance-programme-antimicrobial-utilisation-and-resistance-espaur-report

